# Auricular Perichondritis after a “High Ear Piercing:” A Case Report

**DOI:** 10.21980/J8WH16

**Published:** 2021-04-19

**Authors:** Diego Federico Craik Tobar, Adeola Adekunbi Kosoko

**Affiliations:** * McGovern Medical School at the University of Texas Health Science Center at Houston, Department of Emergency Medicine, Houston, TX

## Abstract

Body piercings in sites other than the ear lobe are becoming increasingly popular. It is not uncommon for patients to present to the emergency department with complications resulting from body piercings. We present a 29-year-old female who underwent a “high ear piercing” which resulted in auricular perichondritis. Left untreated, this infection can progress and potentially result in permanent deformity of the external ear or invasive infection of the surrounding structures. It is important to properly diagnose auricular perichondritis, because unlike many more common soft tissue infections, which are usually due to common skin flora and are simply treated with anti-streptococcal or anti-staphylococcal antibiotics, auricular perichondritis is most commonly due to *Pseudomonas aeruginosa*. The treatment of acute auricular perichondritis specifically requires anti-pseudomonal antibiotic therapy, usually a fluoroquinolone, to avoid inflammatory and cosmetic complications. This case report focuses on the clinical diagnosis of auricular perichondritis, which can be easily misdiagnosed or mistreated on initial assessment in an emergency setting.

**Topics:**

Auricular perichondritis, ear piercing, cartilaginous piercing, otalgia.[Fig f1-jetem-6-2-v30][Fig f2-jetem-6-2-v30]

**Figure f1-jetem-6-2-v30:**
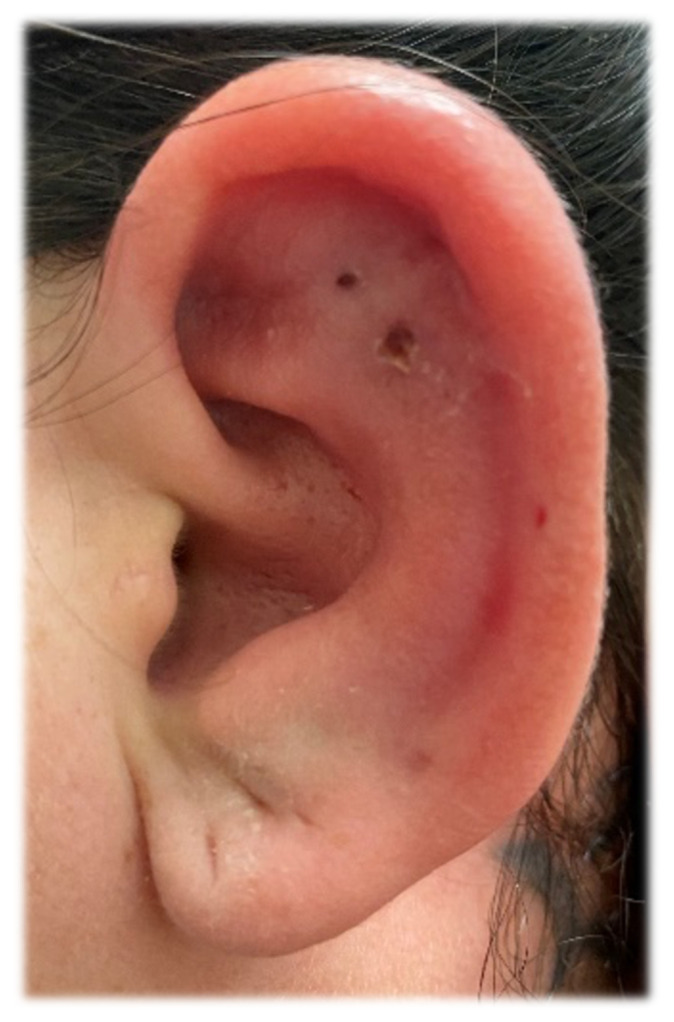


**Figure f2-jetem-6-2-v30:**
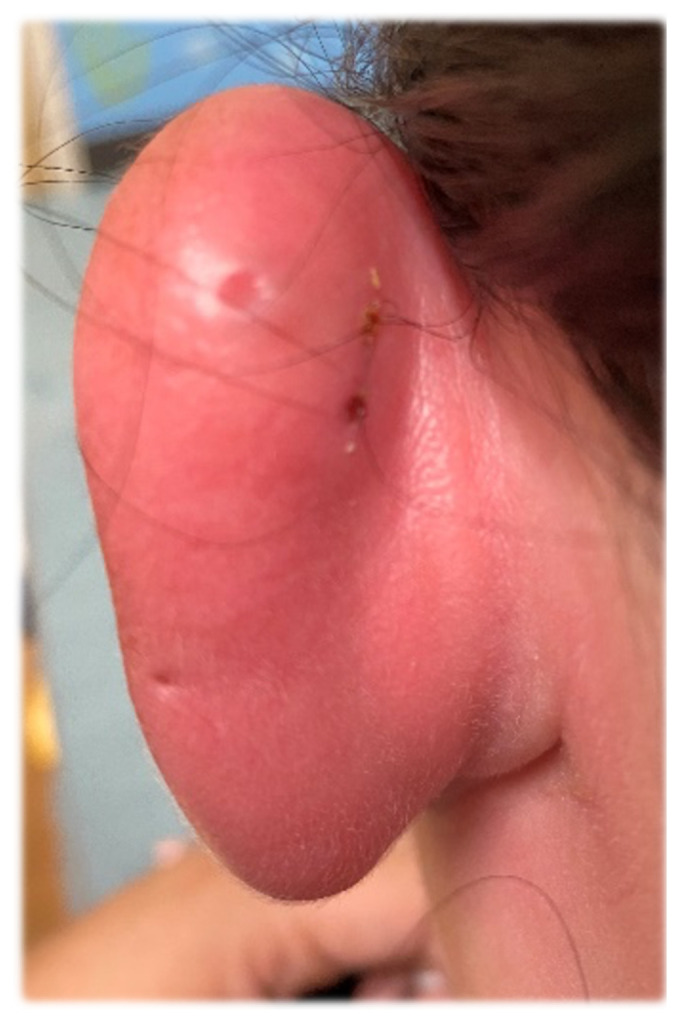


## Brief introduction

Body piercings are common in children and adults. It is not uncommon for patients to present to the emergency setting with complications due to various body piercings, including pain, allergic reactions, bleeding, hematoma, trauma, and infection, among others. This case report describes a patient who underwent a “high ear piercing” resulting in auricular perichondritis. We highlight the importance of early recognition and treatment of chondral infections of the ear.

## Presenting concerns and clinical findings

A 29-year-old woman with no significant medical history presented to the emergency department (ED) with a 2-day history of swelling and severe pain at her left ear. The patient reported that on the day prior to the onset of symptoms, she had visited a newly opened piercing shop and had the upper lateral scapha (the groove between the helix and the antihelix) of her left ear pierced. The next day, she started to notice swelling and redness of her external ear at the site of the new piercing. Subsequently, she developed severe pain in response to touching the ear.

One day prior to presentation to the ED, her husband helped to remove the new piercing using pliers to gain traction around the increasing swelling. Despite over-the-counter analgesics, the pain was intolerable. There was no discharge from the ear or the piercing wound. She described a small, tender bump where her ear met her face, though this bump was not as tender as the area surrounding the piercing site. She denied hearing loss, neck pain, fever, or any other systemic symptoms. The patient initially visited an urgent care center where the nurse practitioner recommended that she go to an emergency center for computer tomography to ensure that she did not have masoiditis.

## Significant findings

On physical examination, there was erythema, swelling, warmth, and general exquisite tenderness of the superior aspect of the left pinna (the outer ear) but excluding the ear canal, lobe, tragus, and crus. There was no facial involvement. There was no fluctuance about the ear and no drainage of fluid. The preauricular lymph nodes were enlarged and tender, but the anterior cervical lymph nodes were not tender. There was no mastoid tenderness, protrusion of the ear, or interruption of the postauricular crease.

## Patient course

We made a bedside diagnosis of acute auricular perichondritis. A complete blood count revealed an elevated white blood cell count of 12.2 × 10^3^ cells/μL with 80.8% neutrophils. Because there were no signs of deep space infection or auricular abscess, the patient was discharged home with 750 mg oral levofloxacin daily for 7 days.

We performed a follow-up phone call with the patient seven days later. She reported having completed the prescribed course of antibiotics. She explained that after discharge, she required over-the-counter analgesics for pain relief and that the ear swelling and redness did not decrease significantly until after 3 or 4 days into the course of therapy. On completion of her course of antibiotics, she experienced complete resolution of erythema, swelling, and pain in her left pinna without any obvious deformity.

## Discussion

The earlobe is the most common site for decorative piercings. However, body piercings and multiple ear piercings beyond the earlobe have recently increased in popularity.^1^,^2^ A high ear piercing is defined as one that involves the superior one-third of the pinna cartilage. These piercings require puncturing through a chondral structure, which has a poorer blood supply than the earlobe, which does not have cartilage. The frequently described complications of ear piercings include allergic reactions, auricular perichondritis, embedded earrings, cellulitis, hypertrophic scarring, keloid formation, and traumatic tears.^3^ The predictors of complications include the piercing site, materials used, experience level of the piercer, hygiene, and aftercare.^1^

Auricular perichondritis is an infection of the ear cartilage. Left untreated, the infection can spread to the subperichondrial space and form a subperichondrial abscess. Without appropriate intervention, auricular perichondritis can result in spreading infection or tissue necrosis with permanent cosmetic deformity.^2^,^4^,^5^

Symptoms typically begin hours to days after the piercing takes place. Shearing forces associated with injury to the perichondrium due to a piercing gun may predispose to perichondritis.^4^,^5^ Similarly, poorly made jewelry with structural irregularities may promote infection. The early features of perichondritis include local warmth, erythema, and pain. Typically, erythema will involve the pinna but not the ear lobe because it has no cartilage. The affected region is often quite tender to touch. There may also be associated surrounding lymphadenopathy. If an abscess develops, there may be fluctuance in the affected region. The diagnosis is typically clinical, and advanced imaging or laboratory testing are not routinely indicated.

It is common for practitioners to misdiagnose auricular perichondritis for a simple cellulitis, often resulting in treatment with ineffective antibiotics.^5^–^8^ It is important to distinguish perichondritis from other soft tissue infections because the notable causative pathogens for auricular perichondritis are *Pseudomonas aeruginosa* and *Staphylococcus aureus*.^9^,^10^ Therefore, medical management is most successful with fluoroquinolone antibiotics, which are effective against *Pseudomonas*, methicillin-susceptible *S. aureus* (MSSA), and *Staphylococcus*, including *S. epidermidis*. Ciprofloxacin, an older drug, has been commonly described in the literature for the management of auricular perichondritis.^2^,^4^–^9^ More recently, there have been reports of successful management with levofloxacin,^6^,^10^ a third-generation fluoroquinolone, as was the case with this patient. Further studies will be necessary to evaluate the efficacy of fourth-generation fluoroquinolones (eg, moxifloxacin, gemifloxacin, delafloxacin), which generally have less activity against *Pseudomonas* than ciprofloxacin but have more activity against MSSA, *Enterococcus*, and *Streptococcus*.^12^–^13^ Fluoroquinolones are generally safe, but there are some adverse effects that are commonly associated with fluoroquinolone use, and practitioners should consider these before prescribing. Routine use of fluoroquinolones in children can potentially cause musculoskeletal toxicity (eg, tendinopathies, arthralgias), but the American Academy of Pediatrics recommends that if fluoroquinolones are indicated, oral formulations are preferred to parental administration in children.^14^ Of the severe possible adverse effects of fluoroquinolones, prescribers should consider aortic aneurysm/dissection particularly in at-risk populations.^15^ Other notable, but less severe or common adverse effects of fluoroquinolones include temporary gastrointestinal irritation, central nervous system disturbance (eg, headache, dizziness, peripheral neuropathy), or prolonged QT interval. Often, a short course of fluoroquinolones for perichondritis is one of the cases in which the benefits typically outweigh the risk. In general, fluroquinolones should be avoided in pregnant and breastfeeding women due to risk for cartilage and bone toxicity in fetuses and infants.^16^

Unfortunately, the other options for appropriate antibiotic coverage are generally intravenous formulations (eg, ticarcillin, piperacillin, cefepime, ceftazidime, gentamicin, and tobramycin). If a concomitant abscess is suspected, the patient should receive plastic surgery or an otorhinolaryngology consultation for prompt drainage.

In summary, early and accurate diagnosis of auricular perichondritis and treatment with a fluoroquinolone antibiotic is important for optimal patient outcomes.

## Supplementary Information






