# Supplementary figures and images for: Auricular Perichondritis after a “High Ear Piercing:” A Case Report
Source: J Educ Teach Emerg Med. 2021 Apr 19;6(2):V30–3. doi: 10.21980/J8WH16 (PMC10332785; doi:10.21980/J8WH16)

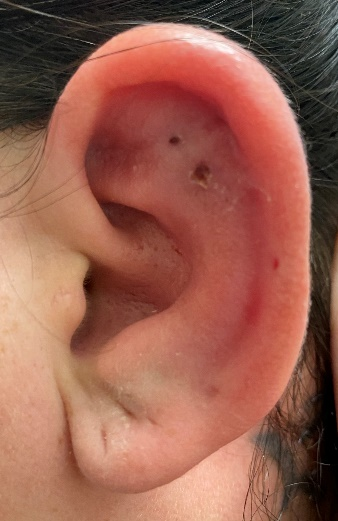

Supplement: Supplementary file 1 [file jetem-6-2-v30-supp1.png]

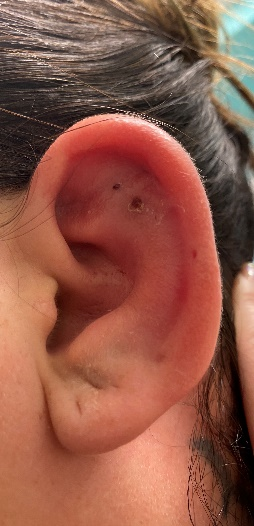

Supplement: Supplementary file 2 [file jetem-6-2-v30-supp2.png]

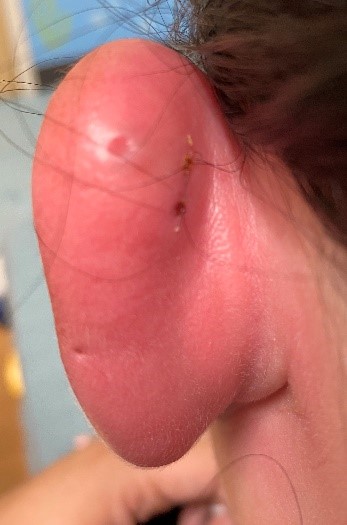

Supplement: Supplementary file 3 [file jetem-6-2-v30-supp3.jpg]
